# T-Cell Redirecting Antibodies for the Treatment of Multiple Myeloma: Off-the-Shelf T-Cell Immunity

**DOI:** 10.1097/PPO.0000000000000847

**Published:** 2026-09-25

**Authors:** Niels W.C.J. van de Donk, Febe Smits, Chloe O’Neill, Jos Hoekman, Sandy Kruyswijk, Serena R. Baglio, Sonja Zweegman, Charlotte L.B.M. Korst

**Affiliations:** * Department of Hematology; † Department of Pathology, Cancer Center Amsterdam, Amsterdam UMC, Vrije Universiteit Amsterdam, Amsterdam, The Netherlands

**Keywords:** multiple myeloma, immunotherapy, T-cells, bispecific antibody, trispecific antibody

## Abstract

Bispecific antibodies (BsAbs) bind simultaneously to an antigen on the surface of multiple myeloma (MM) cells and to CD3 on the surface of T cells. This engagement leads to T-cell activation and degranulation, releasing perforin and granzymes, followed by the killing of the MM cell. Off-the-shelf available BsAbs targeting BCMA, GPRC5D, and FcRH5 have pronounced antitumor activity in heavily pretreated MM with cytokine-release syndrome, neutropenia, and infections as the most common side effects. To further improve clinical outcomes, BsAb-based combinations are being evaluated in earlier treatment lines, including newly diagnosed MM. Notably, the MajesTEC-3 trial established the combination of teclistamab and daratumumab as a new standard-of-care for relapsed/refractory MM as early as first relapse. The most common mechanism underlying acquired resistance to BsAbs is loss of antigen expression; therefore, dual-targeting strategies (combination of BsAbs targeting different tumor antigens or trispecific antibodies) are being intensively investigated to enhance the depth and duration of response.

Treatment options for multiple myeloma (MM) patients have greatly improved over the last few years, particularly with the introduction of immunomodulatory drugs (IMiDs; thalidomide, lenalidomide, and pomalidomide) as well as proteasome inhibitors (PIs; bortezomib, carfilzomib, and ixazomib) and anti-CD38 monoclonal antibodies (mAbs; daratumumab, isatuximab).^1,2^ These drugs are now used in doublet, triplet, or quadruplet combinations, both for first-line therapy and for the treatment of relapsed disease. Before the introduction of modern T-cell immunotherapies, patients who developed disease refractory to IMiDs, PIs, and anti-CD38 mAbs experienced a very poor survival.^1^ This stimulated the development of new anti-MM agents with novel mechanisms of action, such as T-cell redirecting bispecific antibodies (BsAbs) and CAR T-cell therapies. In contrast to CAR T-cell therapies, bispecific antibodies are ‘off the shelf’ therapies, rapidly available to patients without manufacturing delays.^3^

In this review, we will discuss the efficacy and safety of BsAbs, either approved or in advanced stages of clinical testing in MM. We will also discuss how a better understanding of resistance mechanisms has led to the development of new, effective combination regimens and new antibody formats, such as trispecific antibodies (TsAbs).

## MECHANISM OF ACTION OF BISPECIFIC ANTIBODIES

**FIGURE 1:**
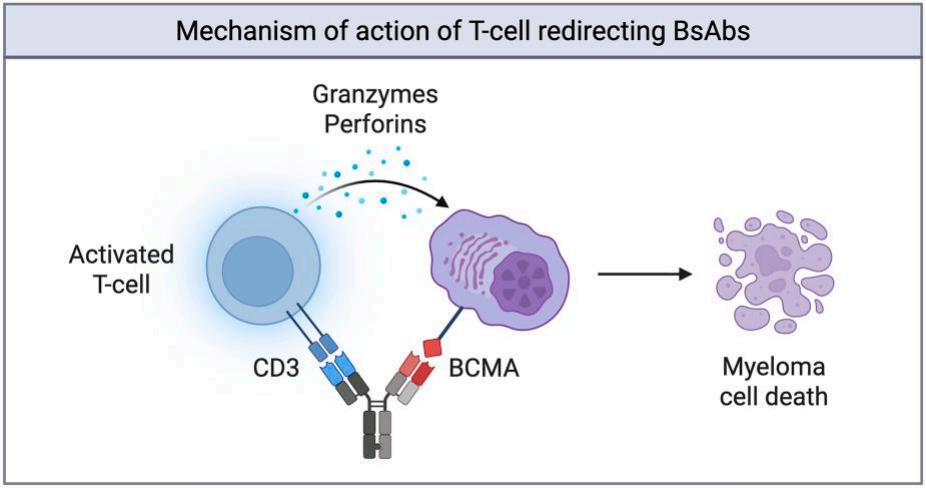
Mechanism of action of T-cell redirecting bispecific antibodies. Bispecific antibodies (BsAbs) bind simultaneously to an antigen on the surface of MM cells and to CD3 on the surface of T cells. This engagement leads to T-cell activation and degranulation, releasing granzymes and perforin, and subsequent killing of the MM cell.

Bispecific antibodies (BsAbs) are designed to simultaneously bind to an antigen on the surface of MM cells and to CD3 on the surface of T cells. This engagement leads to activation and degranulation of the T-cell with release of granzymes and perforins and subsequent killing of the MM cell (Fig. 1).^4,5^

## EFFICACY OF BISPECIFIC ANTIBODIES

### BCMA-Targeting BsAbs

BCMA is highly expressed on normal and malignant plasma cells, as well as on B-cell subsets, (mature B cells, and B-cell precursors [from the small pre-B-cell stage onwards]), making it an attractive target for immunotherapy.^4,6,7^ BsAbs targeting BCMA have demonstrated substantial efficacy in heavily pretreated MM. There are currently 3 BCMA-targeting BsAbs approved by the FDA and EMA for the treatment of triple-class-exposed relapsed/refractory MM patients: teclistamab, elranatamab, and linvoseltamab (Table 1).^8,10,11,13–15^ In the registration studies, these BsAbs induced comparable response rates (ORR: 61%-71%; CR rate: 37%-52%).^8–11^ The median progression-free survival (PFS) was 11.4 months with teclistamab, 17.2 months with elranatamab, and not yet reached with linvoseltamab (18 mo PFS: 60.4%).^8–11^ The lower PFS with teclistamab can be explained by the impact of the COVID-19 pandemic on the MajesTEC-1 trial, which enrolled patients during its peak.^13^ Indeed, when censored for COVID-19 deaths, median PFS with teclistamab was 15.1 months.^16^ Although the efficacy of these BsAbs is comparable, there is a difference in mode of administration: teclistamab and elranatamab are administered via the subcutaneous route, while linvoseltamab is administered intravenously.^8–11^

**TABLE 1:** Efficacy and Safety of Approved BCMA- and GPRC5D-Directed BsAbs

Drug name	Teclistamab, n (%)	Elrananatamab, n (%)	Linvoseltamab, n (%)	Talquetamab, n (%)*
Target	BCMA	BCMA	BCMA	GPRC5D
Type of agent	BsAb	BsAb	BsAb	BsAb
Study	MajesTEC-1^8,9^	MagnetisMM-3^10^	LINKER-MM1^11^	MonumenTAL-1^12^
Dose	1.5 mg/kg SC, after 2 step-up doses	76 mg SC, after 2 step-up doses	200 mg IV, after 2 step-up doses	800 µg/kg SC, after 3 step-up doses
Patient population	TCE, ≥3 prior lines of therapy	TCE	TCE, ≥3 prior lines of therapy	TCE, ≥3 prior lines of therapy
Median number of prior lines	5	5	5	4.5
Triple-class-refractory	77.6	96.7	82.1	71
Number of patients	165	123	117	154
Median follow-up (mo)	30.4	28.4	21.3	19.4
Number of previous lines of therapy (median)	5	5	5	4.5
≥PR	63	61	71	69
≥CR	46	37	52	40
Median PFS (mo)	11.4	17.2	NR, 18 mo PFS: 60.4	11.2
Median OS (mo)	22.5	24.6	NR, 18 mo OS: 69.8	NR, 12 mo OS: 77
CRS: all grade/grade ≥3	72/1	58/0	46.2/0.9	74/1
Neurotoxicity: all grades/grade ≥3	3/0	5/0	7.7/2.6	10/3
Infections: all grade/grade ≥3	79/55	71/49	75.2/36.8	68/18

* Two RP2Ds: 400 µg/kg QW and 800 µg/kg Q2W; data for 800 µg/kg Q2W are provided in the table.

TCE indicates triple-class exposed; IV, intravenous; NR, not reached; SC, subcutaneous.

Other BCMA-targeting BsAbs, such as etentamig (ABBV-383) and MK-4002 (HPN217), are in advanced stages of clinical testing.^17,18^

### GPRC5D-Targeting BsAbs

GPRC5D is expressed on malignant plasma cells, but at substantially lower levels on normal B cells and normal plasma cells.^5^ GPRC5D is also expressed on cells that produce keratin.^19^ Talquetamab is a GPRC5D-targeting bispecific antibody approved by EMA and FDA for the treatment of triple-class-exposed MM patients (Table 1).^12^ At the recommended phase 2 dose of 800 µg/kg Q2W, the ORR was 69% (≥CR: 40%) with a median PFS of 11.2 months.^12^ LBL-034 is another GPRC5D-targeting BsAb that is being evaluated in an ongoing phase 1/2 study for relapsed/refractory MM (RRMM).^20^ Development of forimtamig was stopped (business decision).^21^

### FcRH5-Targeting BsAbs

FcRH5 is expressed across the B-cell lineage, including plasma cells.^22^ Cevostamab, which targets FcRH5, is currently being evaluated in heavily pretreated MM patients.^23^ At the 160 mg target dose with intravenous administration, the ORR was 60.6% (≥CR: 26.8%) in patients who did not previously receive prior BCMA-directed therapies. The median duration of response was 10.4 months.^23^ A recent study showed that cevostamab can also be administered via subcutaneous administration with clinically meaningful responses at target doses of ≥120 mg, with more pronounced activity in BCMA-naïve patients.^24^

## TOXICITY

Bispecific antibodies targeting BCMA, FcRH5, and GPRC5D share toxicities related to T-cell activation (CRS and ICANS), but their on-target/off-tumor toxicity profiles are distinct and depend on the expression of the target antigen in healthy tissues.^25^

### Cytokine-Release Syndrome

The most common adverse effect of BsAb treatment is probably cytokine-release syndrome (CRS). BsAb-induced T-cell activation and subsequent activation of other immune cells (eg, monocytes) result in elevated levels of cytokines such as IL-6, IL-10, IFN-γ, TNF-α, and GM-CSF.^3^ The increase in cytokines may result in a systemic inflammatory response syndrome (CRS) characterized by fever, and in more severe cases, also hypotension and hypoxia.^3,9,13,26,27^ Treatment of CRS with steroids or the IL-6 receptor-blocking antibody tocilizumab is effective, with rapid symptom resolution.^3,26,28^

**FIGURE 2:**
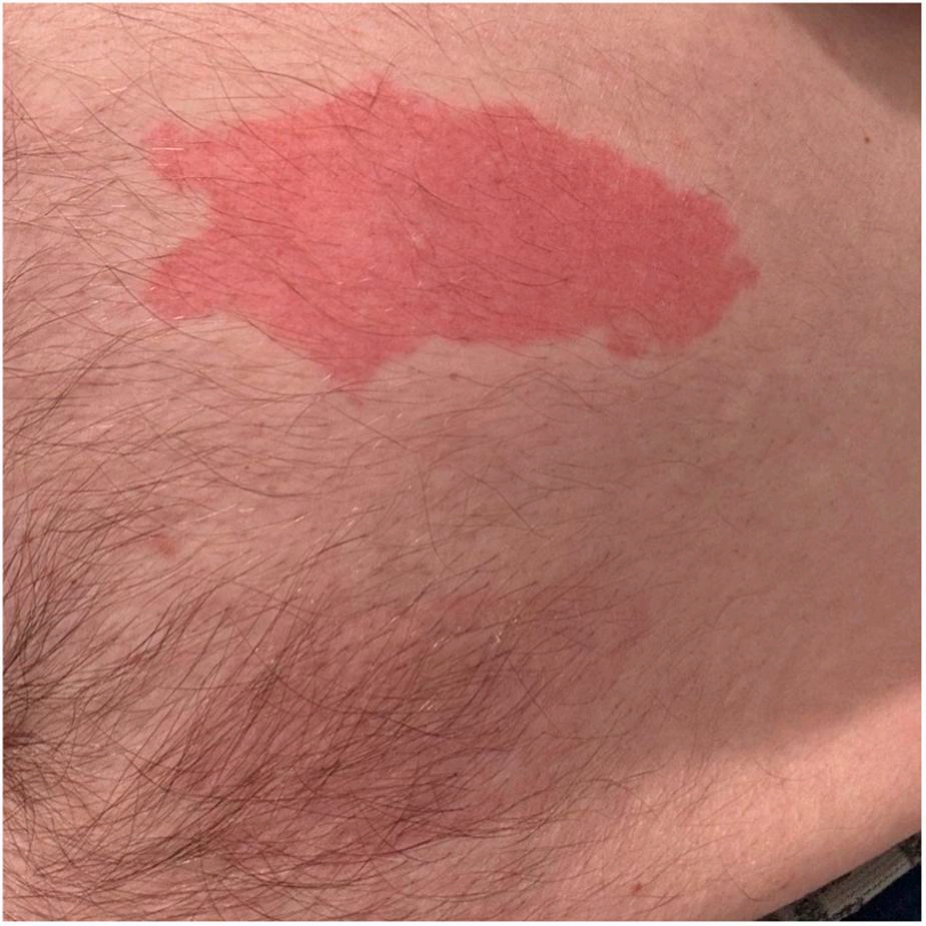
Local administration-related reaction following administration of teclistamab. Local skin inflammation after subcutaneous administration of teclistamab. The inflammation resolved spontaneously a few days after the injection.

CRS mitigation strategies include step-up dosing and premedication with steroids and antihistamines. Patients are often hospitalized to administer step-up doses and the first full dose, allowing adequate monitoring for early signs and symptoms of CRS. Importantly, prophylactic tocilizumab substantially reduces the rate of CRS, thereby enabling outpatient dosing rather than hospitalization.^29–32^ BsAbs with a low CD3 binding affinity also have a lower rate of CRS (eg, CRS rate with etentamig with 1 step-up dose: 23%).^33^ Cytokine production at the injection site may explain local skin inflammation, which can be observed with subcutaneously administered BsAbs (Fig. 2).

### Immune Effector Cell-Associated Neurotoxicity Syndrome

Immune effector cell-associated neurotoxicity syndrome (ICANS) is another adverse event mediated by cytokines secreted by activated T cells and other immune cells.^3^ ICANS is characterized by symptoms such as delirium, confusion, aphasia, seizures, and cerebral edema. It should be realized that severe ICANS is a rare adverse event (<3%) with BsAbs, while it is more common with CAR T-cell therapy. The primary treatment for ICANS is high-dose corticosteroids.^3^

### Infections

Patients receiving BsAbs are at risk of developing infections. BCMA-targeting BsAbs are associated with a higher risk of infections,^13–15^ compared with GPRC5D-targeting BsAbs.^12,34^ This can be explained by the observation that BCMA-targeting BsAbs eliminate normal B-cell and polyclonal plasma cells, resulting in severely suppressed levels of IgG, IgA, and IgM.^6,7^ Hypogammaglobulinemia increases the susceptibility to (encapsulated) bacterial infections as well as viral infections.^6^ GPRC5D-targeting BsAbs spare normal B cells and polyclonal plasma cells, which explains why there is no decrease in polyclonal immunoglobulin levels.^35^ In fact, IgG levels may even increase in patients who experience a sustained response with talquetamab.^35^ Cevostamab also appears to have a lower risk for infections compared with BCMA-targeting BsAbs (all-grade infections: 54.5%; ≥grade 3: 19.2%).^23^ This may be related to the observation that cevostamab does not lead to persistent B-cell depletion.^36^

In addition to impacting humoral immunity, BCMA-, FcRH5-, and GPRC5D-targeting BsAbs may induce neutropenia, which may contribute to the development of bacterial infections. In addition, continuous use of BsAbs resulting in sustained T-cell stimulation is associated with reduced T-cell fitness.^37^ This may explain the occurrence of opportunistic infections associated with T-cell dysfunction, such as CMV-related diseases (eg, colitis or retinitis), progressive multifocal leukoencephalopathy caused by JC virus, or pneumocystis jirovecii pneumonia (PJP).^13^

Infectious risk can be reduced by IgG supplementation in patients with low IgG levels (mandatory for patients with BCMA-targeting BsAbs; infrequently needed in patients treated with GPRC5D-targeting BsAbs).^6,38^ Neutropenia can be managed with granulocyte colony-stimulating factor (G-CSF) support.^25^ All patients treated with BsAbs should also receive PJP prophylaxis and varicella zoster prophylaxis.^25^ Patients who are frail or have sustained neutropenia (which is rare with BsAbs) might also benefit from anti-bacterial prophylaxis (eg, levofloxacin during the first 1-2 cycles).^25^ Several studies are investigating maintenance strategies or fixed-duration BsAb treatment to reduce the risk of infections.^39–41^

### On Target and off Tumor Adverse Events

**FIGURE 3:**
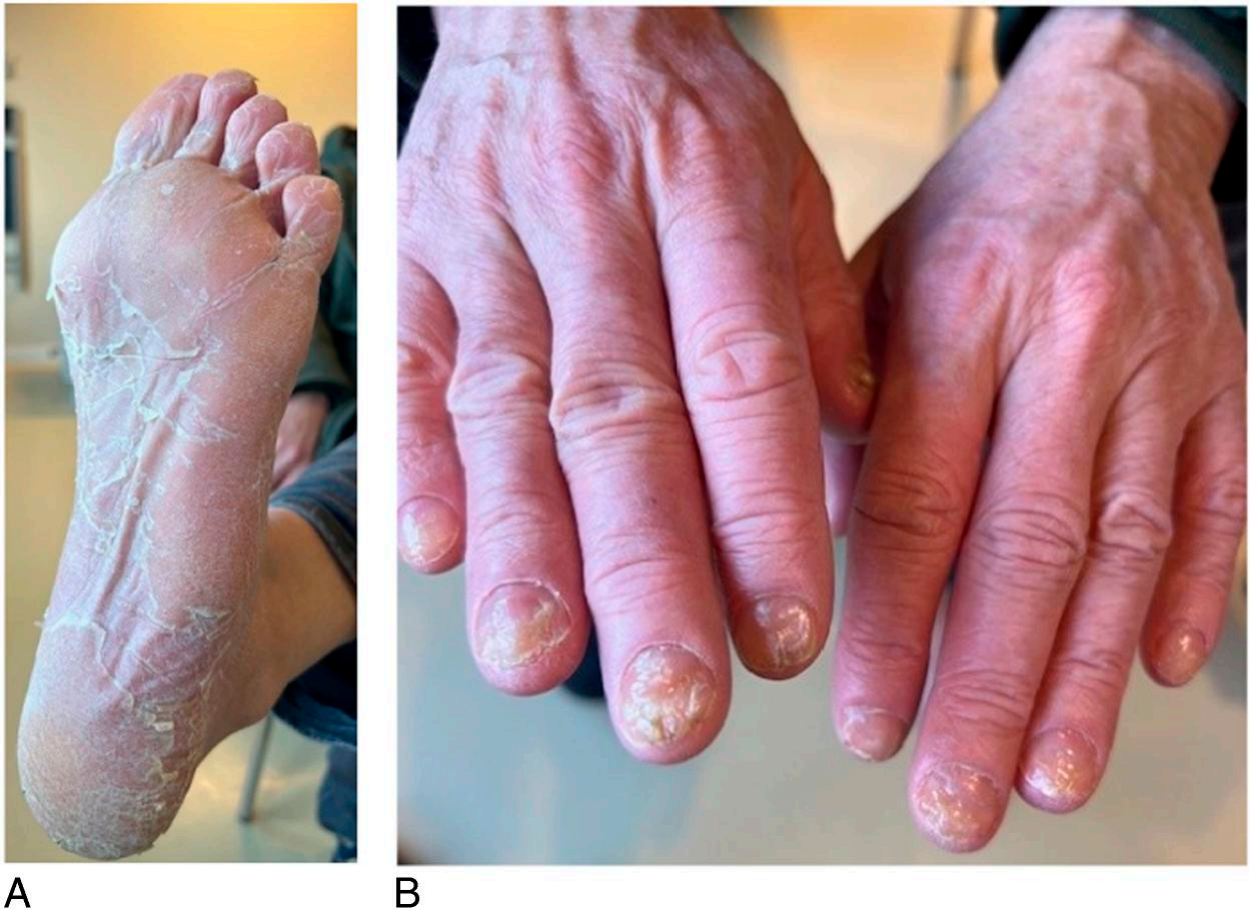
On target/off tumor adverse events associated with treatment with GPC5D-targeting BsAbs (A): desquamation of the foot soles, occurring early after initiation of talquetamab treatment. B, Nail changes observed in a patient treated with talquetamab. The patient experienced benefits from a nail hardener.

In the section on infections, we already discussed that the depletion of normal B cells and normal plasma cells is an on-target/off-tumor effect of BCMA-targeting BsAbs, but not of GPRC5D-targeting BsAbs.^6,35^ However, treatment with GPRC5D-targeting BsAbs frequently results in specific adverse events such as skin and nail disorders (Fig. 3), as well as oral adverse events, which are related to expression of the target antigen in healthy tissues (eg, hair follicles, nails, and filiform papillae of the tongue).^19^ Exfoliation of the skin over hand palms and foot soles is a common adverse event (Fig. 3A), while rash is less common with talquetamab.^19^ Skin toxicities can be effectively managed with emollients and, in case of an inflammatory component, with topical corticosteroids or oral corticosteroids.^19^ Nail toxicities include onycholysis (separation of the nail from its nail bed), onychomadesis (detachment of the nail from its nail bed), onychoclasis (nail breakage), and nail ridging (Fig. 3B).^19^ Some patients benefit from nail hardeners.

Talquetamab is also associated with oral toxicities such as dysgeusia and dysphagia, which can lead to weight loss.^19,42^ Importantly, taste loss is reversible, especially after increasing the interval between administrations in patients who have achieved deep response.

Although rare, patients treated with talquetamab may also develop dizziness, ataxia, dysarthria, and/or nystagmus. The underlying cause of these symptoms remains incompletely elucidated, but it may be related to GPRC5D expression in the inferior olivary nucleus (a structure in the medulla oblongata that relays motor and sensory signals from the spinal cord to the cerebellum and regulates motor coordination).^43^

## RESISTANCE MECHANISMS

∼30% to 40% of patients do not respond to BsAb treatment. Primary refractory disease can be related to the presence of a high tumor burden with an unfavorable effector-to-target ratio or the presence of extramedullary disease.^44^ Several studies have also shown that poor T-cell fitness at baseline or high numbers of immune suppressor cells (eg, regulatory T cells) are associated with nonresponse to BsAbs.^45,46^ In addition, most patients eventually relapse after BsAb treatment.^44,45^ The most frequent cause of disease progression after initial response is probably loss of target expression due to genetic or epigenetic alterations. In a large cohort of patients who relapsed during BCMA-targeting BsAb treatment (n=31), the rate of BCMA antigen escape was 64.5%.^47–49^ Similarly, GPRC5D antigen escape is a key driver of relapse during GPRC5D-targeting BsAb therapy.^47,49–52^ In a single-center study evaluating talquetamab treatment (n=18), 83% of patients lost GPRC5D expression (as determined by immunohistochemistry) at the time of disease progression.^52^ There are currently no data available on how frequently FcRH5 loss occurs during treatment with cevostamab.

## COMBINATION STRATEGIES IN HEAVILY PRETREATED MULTIPLE MYELOMA

To improve response rate and response duration, several combination strategies and dual targeting have shown promising results.

### Immunomodulatory Drugs

**FIGURE 4:**
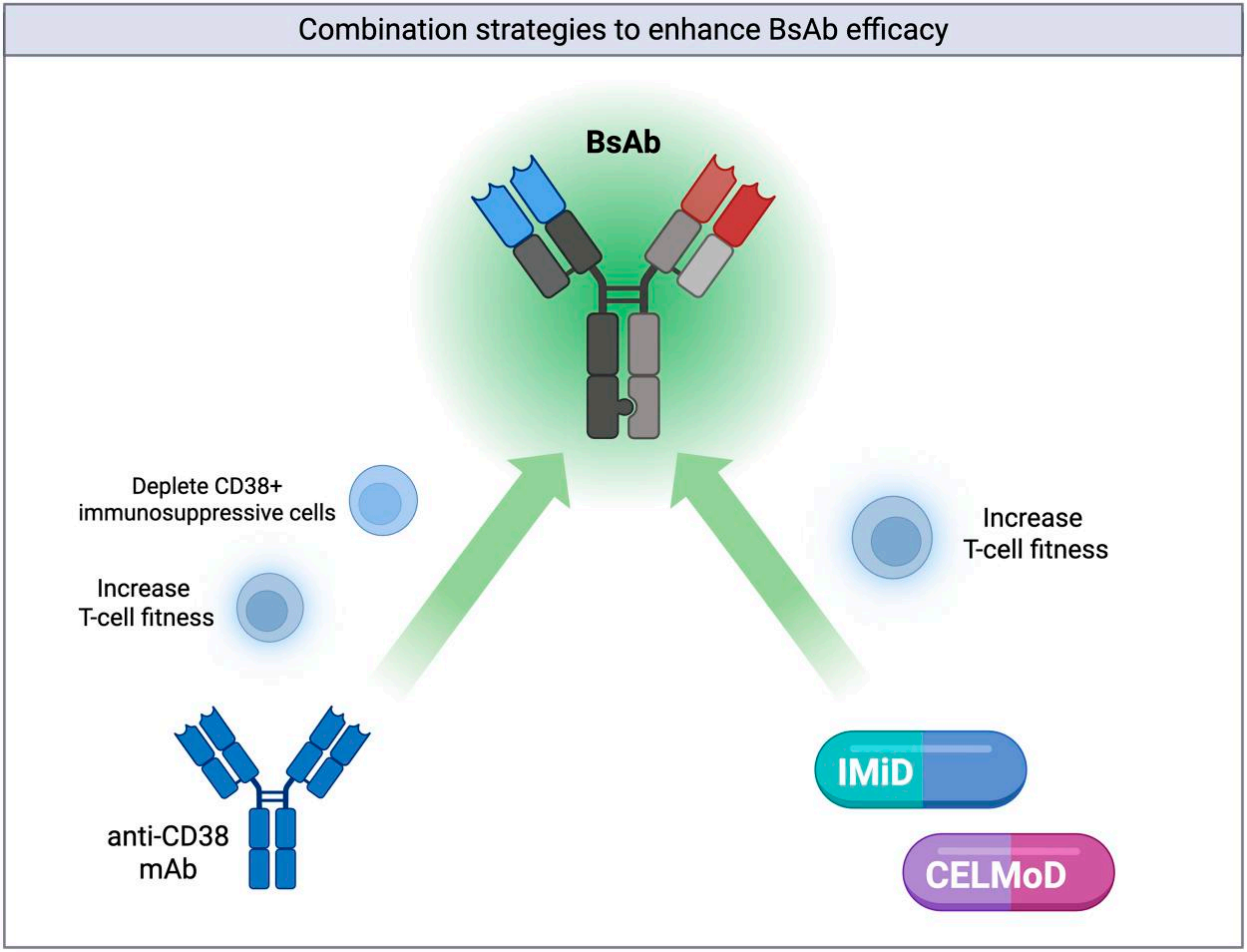
Combination regimens with T-cell redirecting bispecific antibodies. Both CD38-targeting antibodies and IMiDs/CELMoDs enhance the activity of BsAbs through their direct antitumor and T-cell stimulatory effects.

IMiDs have multiple mechanisms of action, including direct antitumor activity and immunostimulatory effects (Fig. 4).^53^ Lenalidomide is being evaluated as a partner drug with BsAbs in large phase 3 trials in newly diagnosed MM.^54^ In the relapsed/refractory setting, pomalidomide is a promising combination partner for BsAbs.^33,36^ Iberdomide and mezigdomide (Cereblon E3 Ligase modulatory drugs; CELMoDs) have superior immune-stimulating effects compared with IMiDs.^55^ The superior immune stimulation with CELMoDs can be explained by their enhanced ability to induce conformational changes in cereblon (transition from the open to the closed conformation), resulting in improved recruitment and degradation of substrates such as Aiolos and Ikaros, compared with IMiDs.^55,56^ In the phase 1 MagnetisMM-30, elranatamab combined with iberdomide demonstrated a favorable safety profile and encouraging efficacy in 22 patients who received 2 to 4 prior lines of therapy.^57^ At a median follow-up of 7.8 months, the ORR was 95.5% (≥CR: 45.5%).^57^ As expected, the addition of iberdomide increased the rate of grade 3/4 neutropenia (72.7%).^57^ Various studies are also exploring mezigdomide in combination with a BsAb.^58^

### CD38-Targeting Antibodies

CD38-targeting antibodies (daratumumab and isatuxumab) have multiple mechanisms of action, including direct antitumor activities [eg, antibody-dependent cellular cytotoxicity (ADCC), complement-dependent cytotoxicity (CDC), and antibody-dependent cellular phagocytosis (ADCP)].^2^ In addition, CD38-targeting antibodies also have immunomodulatory effects by eliminating CD38^+^ immunosuppressive cells, such as regulatory T cells (Tregs), regulatory B cells (Bregs), and myeloid-derived suppressor cells (MDSCs), resulting in the expansion and activation of CD4^+^ and CD8^+^ T cells (Fig. 4).^59^

The TRIMM-2 study provided the first evidence that daratumumab may enhance the activity of BCMA- and GPRC5D-targeting BsAbs, even in patients with daratumumab-refractory disease.^60,61^ The phase 3 MajesTEC-3 trial was initiated on the basis of these promising results to evaluate teclistamab plus daratumumab in early relapsed/refractory MM (see also below in the section “earlier lines of therapy”).

### Dual-Antigen Targeting Strategies

As described above, antigen escape appears to be the most common mechanism of relapse after initial response with a BCMA- or GPRC5D-targeting BsAb. Dual antigen targeting is a novel strategy to prevent antigen-negative escape. Several dual-targeting therapies are being evaluated in the clinic, including combinations of 2 different BsAbs and TsAbs.

The combination of 2 BsAbs targeting different tumor antigens (BCMA with Teclistamab and GPRC5D with talquetamab) was evaluated in the RedirecTT-1 study.^62^ This combination demonstrated deep and durable responses in patients with relapsed/refractory disease, also in patients with extramedullary disease who typically have very poor survival with BsAbs.^62^ The promising results in patients with extramedullary disease resulted in the initiation of a phase 2 study exclusively for patients with extramedullary disease.^63^ With a median follow-up of 16.8 months, the ORR was 79% (≥CR: 53%) with a median PFS of 15.0 months.^63^ These results compare favorably to what can be expected from single-antigen targeting BsAbs in patients with extramedullary disease.^12,13,44^

Ramantamig (JNJ-5322) is currently the most advanced TsAb evaluated in clinical trials. Ramantamig simultaneously targets BCMA and GPRC5D on MM cells and CD3 on T cells, with enhanced efficacy compared with mono-targeting controls in preclinical studies.^64,65^ First clinical results are promising, with a 100% ORR rate (≥CR: 88.9%) in 27 patients naïve to BCMA- and GPRC5D-directed therapies, who were treated with the recommended phase 2 dose.^64,65^ MRD-negativity at 10^−5^ and 10^−6^ was 100% in evaluable patients treated at the RP2D. With a median follow-up of 17.8 months, the 12-month PFS was 92.6%.^64,65^ The most frequent adverse events were neutropenia, infections, and skin- and nail-related events, whereas dysgeusia and weight loss were less common compared with the GPRC5D-targeting BsAb talquetamab.^64,65^ Several other BCMAxGPRC5DxCD3 TsAbs are undergoing clinical evaluation.^66,67^

ISB2001 is another T-cell redirecting TsAb that simultaneously targets BCMA and CD38 on tumor cells. Early results from the ongoing phase 1 trial show promising findings (ORR with ≥50 µg/kg: 79%), including in high-risk subgroups, such as patients with extramedullary disease.

The alternative to simultaneously targeting 2 tumor-associated antigens is the sequential use of BsAbs. However, it has been shown that the efficacy of the second BsAb is markedly reduced relative to that of the first BsAb. For instance, in the MonumenTAL-1 study, median PFS with talquetamab was only 3.9 months, compared with 7.5 to 11.2 months in patients not previously exposed to BsAbs.^12^ The reduced efficacy may be explained by reduced T-cell fitness resulting from prolonged T-cell stimulation or development of T-cell-resistant tumors.^37^ Preliminary evidence suggests that a BsAb-free interval of ≥6 months may enhance the efficacy of the second BsAb, possibly by allowing T cells to recover from previous BsAb exposure.^68^

## EARLIER LINES OF THERAPY

### Early Relapse Setting

BCMA- and GPRC5D-targeting BsAbs are evaluated in large, randomized phase 3 studies in the early relapse setting either as monotherapy or in combination with other drugs. T-cell fitness is better in earlier lines of therapy,^69^ and we therefore expect that the efficacy of BsAbs will be superior compared with what has been observed in end-stage MM.

One of these phase 3 studies is the MajesTEC-3 study, which evaluates teclistamab plus daratumumab versus standard-of-care [daratumumab-pomalidomide-dexamethasone (DPd) or daratumumab-bortezomib-dexamethasone (DVd)] in patients who received 1 to 3 prior lines of therapy, including a PI and lenalidomide.^70^ Patients with prior BCMA-directed therapy or refractory to a CD38-targeting antibody were excluded. With a median follow-up of 34.5 months, teclistamab plus daratumumab significantly improved overall response rate (89.0% vs. 75.3%), CR rate (81.8% vs. 32.1%), and PFS (36 mo PFS: 83.4% vs. 29.7%) compared with standard of care.^70^ Overall survival was also superior with teclistamab plus daratumumab compared with the standard of care. Grade 3/4 infections occurred in 54.1% and 43.4% of patients treated with teclistamab plus daratumumab or standard-of care, respectively. Fatal infections were more common with teclistamab plus daratumumab (4.6%) than compared with standard regimens (1.4%). Infectious deaths occurred mostly within the first 6 months after treatment initiation in the absence of IgG supplementation.^70^ Importantly, new onset grade ≥3 infections decreased over time, coinciding with transition to Q4W dosing and supported by appropriate infectious prophylaxis.^70^ Together with BCMA-directed CAR T-cell therapies and belantamab mafodotin-containing regimens, teclistamab+daratumumab represents another highly effective novel BCMA-directed early relapse option.^71^

The MajesTEC-3 study excluded patients with disease refractory to CD38-targeting antibodies. In contrast, the MajesTEC-9 study evaluated teclistamab monotherapy versus standard-of-care [pomalidomide, bortezomib, and dexamethasone (PVd) or carfilzomib and dexamethasone (Kd)] in patients with 1 to 3 prior lines of therapy, including a CD38-targeting antibody and lenalidomide.

A recent press release reported that in this patient population, which was predominantly refractory to anti-CD38 therapy (85%) and lenalidomide (79%), teclistamab treatment resulted in superior PFS (*HR*=0.29) and OS (*HR*=0.60) compared with the standard of care.

### Newly Diagnosed Multiple Myeloma

BsAbs are also being evaluated in patients with newly diagnosed MM. First, several studies are investigating the efficacy of BsAb-based combinations as induction treatment. The phase 2 GMMG-HD10/DSMM-XX/MajesTEC-5 study evaluates BsAb-based induction regimens in transplant-eligible NDMM.^72^ The first results from this study showed that induction therapy consisting of teclistamab combined with daratumumab and lenalidomide with or without bortezomib was highly effective, with all evaluable patients achieving MRD-negativity (10^−5^).^72^

Teclistamab, combined with daratumumab and lenalidomide, was also effective in transplant-ineligible patients, as shown in safety run-in cohort 1 of the MajesTEC-7 study.^73^ Almost all patients responded (ORR: 92.3%; ≥CR: 80.8%) with a 12-month PFS of 92.6% after a median follow-up of 13.8 months.^73^ The ongoing phase 2 TecLille study evaluates teclistamab+daratumumab (without lenalidomide) in transplant-ineligible patients with NDMM.^74^ This study showed a 100% response rate with 100% MRD-negativity (10^−6^) in evaluable patients.^74^ With a median follow-up of 10.3 months, PFS at 12 months was 100%.^74^ Another large frontline trial for transplant-ineligible patients is the MagnetisMM-6 study with elranatamab+daratumumab+lenalidomide versus daratumumab-lenalidomide-dexamethasone.^75^

Interestingly, the LINKER-MM4 study evaluates linvoseltamab as a monotherapy in both transplant-eligible and transplant-ineligible NDMM patients.^76^ Preliminary results at a median follow-up of 9.2 months showed a ORR rate of 86% (≥CR: 43%) with linvoseltamab administered at a dose of 200 mg.^76^ The phase 3 Linker-MM6/EMN39 study will evaluate DRd induction followed by linvoseltamab compared with continued DRd in patients with transplant-ineligible newly diagnosed MM.

Ongoing studies with larger numbers of patients will provide a better understanding of the optimal combination regimen for patients with newly diagnosed MM. High-risk subsets may benefit from 3-drug combination strategies, while “less may be more” for elderly/frail patients.

In addition, several studies are evaluating the value of maintenance therapy with BsAbs after induction therapy to improve response and survival in patients with newly diagnosed MM. The phase 3 MajesTEC-4/EMN30 study evaluates teclistamab with or without lenalidomide versus lenalidomide maintenance therapy after autologous stem cell transplantation in patients with NDMM. Early results from the safety run-in showed that all evaluable patients were MRD-negative (10^−5^) during maintenance.^54^ Similarly, the Immunoplant study showed that a short course of linvoseltamab (4-6 cycles) was highly effective in MRD conversion from positive to negative in patients with NDMM who remained MRD-positive after initial induction treatment [MRD-negativity rate (10^−6^): 100%].^77^ Notably, in all frontline trials, infections were frequently observed with all studies showing the importance of adequate infectious prophylaxis, including IgG supplementation.^3^

## CONCLUSIONS

BsAbs have substantially improved the survival of patients with triple-class-exposed MM. Given their high efficacy in advanced MM, various large phase 3 trials are now evaluating BsAbs as monotherapy or in combination with other drugs in earlier lines of therapy, including for newly diagnosed MM. The majesTEC-3 study already established teclistamab-daratumumab as a new standard-of-care for RRMM as early as first relapse. We also expect that highly active TsAbs will move into earlier treatment settings and improve survival in patients with multiple myeloma.
